# Cosmic Signs in Radiology: A Pictorial Review

**DOI:** 10.3390/diagnostics15192528

**Published:** 2025-10-07

**Authors:** Yara Jabbour, Raquelle El Alam, Sara Amro, Mutaz Kassas, Youssef Ghosn, Reve Chahine, Mihran Khdhir, Lara Nassar

**Affiliations:** 1 Department of Diagnostic Radiology, American University of Beirut Medical Center, Beirut 1107-2020, Lebanon; 2 Department of Radiology, Brigham and Women’s Hospital, Boston, MA 02115, USA; 3 Faculty of Medicine, American University of Beirut, Beirut 1107-2020, Lebanon; 4 Faculty of Medicine, Jules Bordet Institute, 1 Rue Héger Bordet, 1000 Brussels, Belgium; 5 Department of Radiology, Memorial Sloan Kettering Cancer Center, New York, NY 10065, USA; 6 Department of Radiology, University of Michigan Health System, 1500 E. Medical Center Dr., Ann Arbor, MI 48103, USA; 7 Department of Radiology and Biomedical Imaging, Yale New Haven Hospital, New Haven, CT 06510, USA

**Keywords:** radiological signs, radiographic image interpretation, pattern recognition, radiology interpretation, radiologic diagnosis, cosmic signs

## Abstract

Pattern recognition remains a cornerstone of radiologic interpretation, as it facilitates a confident and comprehensive differential diagnosis. Certain pathologies present with specific and highly recognizable patterns on imaging modalities. These patterns can resemble familiar real-life phenomena, including cosmic bodies that surround us. We present in this article a compilation of radiologic signs across various modalities that take inspiration from cosmic phenomena. For each sign, we summarize its defining imaging appearance, typical clinical context, and common pitfalls; where available, we note diagnostic performance (e.g., sensitivity/specificity) to guide appropriate weighting in practice. By coupling memorable imagery with succinct clinical guidance, this pictorial review aims to support a faster, more accurate pattern recognition that is applicable in both low-resource and tertiary care settings, while recognizing that these signs function as educational aids rather than validated diagnostic tests. In familiarizing themselves with these classic signs, training radiologists can benefit from an engaging and memorable way of recognizing various pathological conditions.

## 1. Introduction

When interpreting findings on imaging, radiologists rely on their sense of pattern recognition to establish a comprehensive differential diagnosis. These patterns can come together to resemble objects or phenomena encountered in everyday life, as was described in several previous review articles discussing radiological signs [1,2,3,4].

We present in this pictorial review a group of radiological disease manifestations resembling cosmic and celestial bodies. In providing a broad collection of classic signs, we aim to assist training radiologists in the recognition and diagnosis of a multitude of pathologies. Our use of vivid, metaphoric signs aligns with the findings of a randomized trial demonstrated that teaching radiology using metaphoric (pareidolic) signs significantly improved short-term knowledge retention and student engagement compared to traditional anatomical correlation [5]. The choice of cosmic patterns provides a unique and widely recognizable theme, but is ultimately one illustrative framework alongside others, rather than a superior approach.

The study draws on cosmic signs identified as most relevant and educational by experienced radiologists and trainees, complemented by a literature review on the topic. While it does not aim to systematically list all possible radiological features, it emphasizes those with the highest value for understanding, retention, and cross-modality comparisons. We identified several signs on different imaging modalities, including ultrasonography, conventional radiographs, computed tomography, magnetic resonance imaging, and angiography, spanning all fields of radiology. This pictorial review used purposive sampling to maximize educational value across modalities and organ systems. We included signs that (i) have a peer-reviewed description and accepted usage; (ii) offer diagnostic value (e.g., characteristic pattern, specificity, or management implications) or highlight a common artifact/pitfall important for trainees; (iii) exhibit a clear and teachable resemblance to a celestial phenomenon; and (iv) could be illustrated with high-quality, de-identified institutional images.

For every sign, we included an image from the database of our institution with a description of the pathology, coupled with an illustration of the corresponding sign seen in nature to highlight the resemblance between the two. With the exception of one sign, our cases derive from a single-institution retrospective archive, which may limit their generalizability to other contexts; however, the signs are shown for their characteristic appearance rather than to imply population prevalence or representativeness. All images were acquired on modern clinical scanners using departmental standard protocols routinely applied in clinical practice; details of the specific acquisition parameters of each radiological image displayed in this manuscript were not included as the images were obtained over several years on multiple different clinical scanners from different vendors with scanner-dependent standard settings.

## 2. Cosmic Signs

### 2.1. Comet-Tail Artifact

#### 2.1.1. On Ultrasound (US)

**Figure 1:**
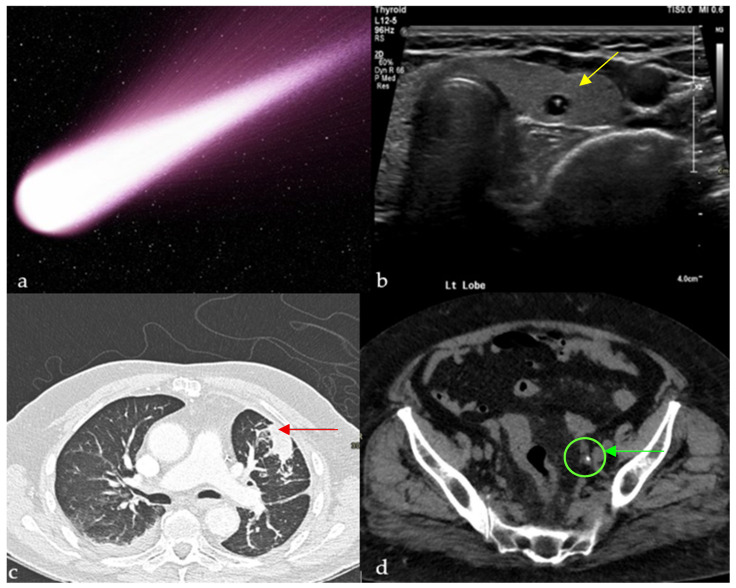
Small calcific/crystalline highly reflective structure causing reverberation artifact on ultrasound of the thyroid (yellow arrows) (**b**), axial CT of the chest showing a curvilinear opacity extending from a subpleural mass toward the ipsilateral hilum with distortion of vessels and bronchi (red arrow) (**c**), and tail of soft tissue extending from a calcification, representing the collapsed/scarred/thrombosed parent vein on a non-enhanced CT of the pelvis (green arrow) (**d**), all resembling a comet tail (**a**).

Similarly to a comet-tail (Figure 1a), this reverberation artifact appears as a sequence of parallel echogenic bands emanating from a definitive source [6]. It is attributed to reverberations from adjacent, highly reflective surfaces. Clinically, it clues into the diagnosis of multiple pathologies, such as adenomyomatosis of the gallbladder (due to cholesterol crystals within Rokitansky-Aschoff sinuses [7]) and colloid nodules in the thyroid (Figure 1b) [8]. It is a reassuring sign during focused assessment with sonography in trauma (FAST) examinations, since its appearance decreases the likelihood of a pneumothorax, with reported sensitivity and negative predictive value approaching 100%, albeit with a specificity of around 60% in differentiating from other interstitial processes [9,10]. This artifact is also present with calcifications and foreign bodies, such as clips and intrauterine devices [8].

A recent case report describes comet-tail reverberation artifacts in the urinary bladder caused by cholesterolic debris in a patient with acute pancreatitis and Type V hyperlipidemia, highlighting that this artifact may also signal systemic lipid metabolism abnormalities beyond its classic associations with gallbladder adenomyomatosis or thyroid colloid nodules [11].

#### 2.1.2. On Computed Tomography (CT)

##### Chest

This finding can be appreciated on both conventional chest radiographs and CT scans [12]. It represents round atelectasis (the comet’s nucleus), a benign form of pulmonary collapse adjacent to the pleural surface, which can mimic a malignancy, hence its other name, “pulmonary pseudotumor” [13]. This folding results in traction on the bronchovascular bundle, which leads to the typical curvilinear opacity originating from the mass and pointing towards the ipsilateral hilum (Figure 1c) [12]. As for the origin of these nodules, some attribute it to a local cleft in the visceral pleura resulting from an underlying effusion, others to a local process causing pleuritis, such as in the case of asbestosis [12]. Round atelectasis with the comet-tail sign is often associated with patients who have a history of chronic pleural disease, prior effusion, or asbestos exposure, and may present with cough, dyspnea, or pleuritic chest pain, though many cases remain asymptomatic and are discovered incidentally. Relying on radiological signs such as this one to differentiate this benign entity from malignancies is crucial because it saves patients unnecessary invasive procedures [13].

##### Abdomen and Pelvis

The clinical usefulness of this sign mainly lies in distinguishing a ureteric stone from a phlebolith, more notably in the anatomic pelvis [1]. The hyperdense calcified phlebolith is thought to resemble the body of the comet, whereas the remaining non-calcified vein would be the tail (Figure 1d). It is important to note that the combined secondary signs of obstructions (asymmetric stranding of perinephric fat, hydroureter, and hydronephrosis [14]) remain the clues with the highest positive predictive value to establish the presence of an obstructing stone [15]. In fact, studies showed the low reliability of the comet-tail sign in ruling out ureteric stones, with a sensitivity of 21–65% [16], and that in fact it might contribute to a missed diagnosis in some ambiguous cases [15]. Patients with acute flank pain may have calcifications in the pelvis; the comet-tail sign helps distinguish benign phleboliths from obstructing ureteral stones, guiding appropriate urologic management.

#### 2.1.3. On Magnetic Resonance Imaging

The comet-tail sign in brain metastasis, as observed on MRI, is characterized by a distinct appearance where the lesion appears to have a tail-like structure extending from the primary mass, resembling the tail of a comet. This imaging feature is typically seen in metastases located in the brain parenchyma, particularly those with a central necrotic core. The tail is thought to represent a zone of peritumoral edema or infiltrating tumor cells extending from the mass. The comet tail sign is considered a helpful radiologic feature for differentiating brain metastases from other types of tumors, as it often indicates aggressive tumor behavior with significant surrounding tissue involvement [17].

### 2.2. Earth-Heart Sign

**Figure 2:**
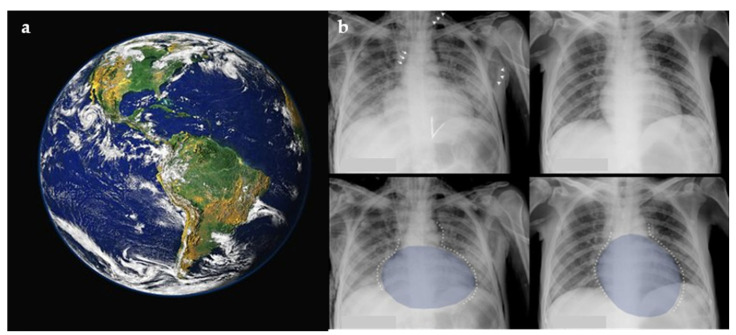
The Earth as it appears from outer space (**a**), with resemblance to the flattening and posterior displacement of the heart by the tension pneumomediastinum on a plain chest radiograph (**b**). Figure used with permission of the author G. A. O. Carillo; *The Lancet*, 2014 [19].

One of the recent additions to the constellation of cosmic signs in radiology is the Earth-Heart Sign, which indicates the presence of a life threatening tension pneumomediastinum [18]. In acute cardiopulmonary deterioration with chest pain and subcutaneous emphysema, recognition of the Earth-Heart configuration on chest radiograph raises concern for tension pneumomediastinum and prompts emergent decompression and airway/ventilatory optimization. The free air leads to a rise in intra-mediastinal pressure and, hence, decreased venous return. This causes flattening of the heart chambers, which increases the transverse cardiac diameter and decreases the vertical cardiac diameter, resembling the earth as seen from outer space (Figure 2a,b) [19].

### 2.3. Galaxy Sign

**Figure 3:**
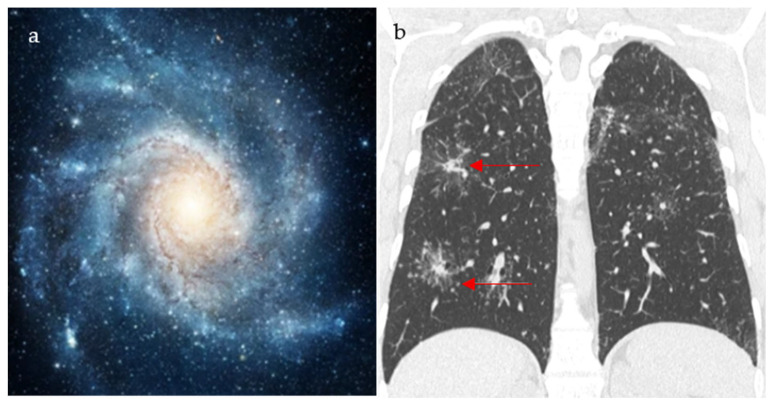
An image depicting a far galaxy (**a**) with resemblance to the CT view of the chest showing mass-like regions composed of numerous smaller granulomas with a central core and peripheral nodules seen in sarcoidosis (red arrows) (**b**).

This sign is seen on chest CT in multiple granulomatous diseases and malignancies, and it is most known to be a feature of pulmonary sarcoidosis [20]. Mimicking the appearance of a galaxy (Figure 3a), it appears as a large pulmonary parenchymal nodule, typically located along the lymphatics, formed by the coalescence of smaller non-caseating granulomas (proven by radiologic-pathologic correlation [21]), with tiny satellite low attenuations nodules more apparent at the periphery, (Figure 3b) [20]. In a Japanese single-center cohort, the galaxy sign on CT had a sensitivity of 23.1% and a specificity of 98.1% for distinguishing pulmonary sarcoidosis from tuberculosis, highlighting its low sensitivity but high diagnostic specificity in appropriate clinical settings [22]. In rare instances, it may be associated with cavitation and ground glass attenuations [21]. In patients with respiratory or systemic symptoms suggestive of sarcoidosis, a galaxy-like nodule composed of confluent granulomas supports the diagnosis and guides further staging and extrapulmonary evaluation.

### 2.4. Loss of Half-Moon Overlap Sign

**Figure 4:**
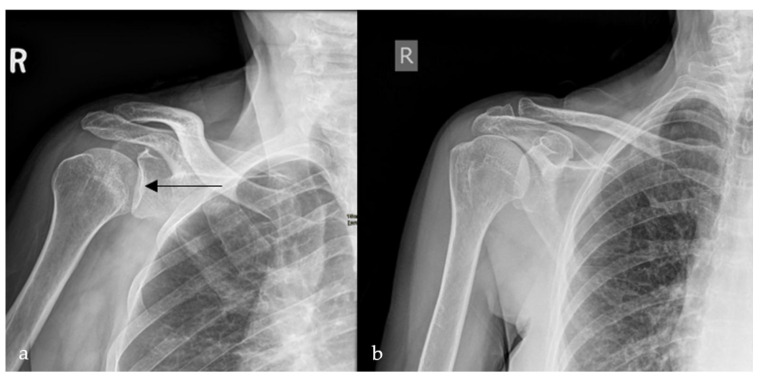
Plain radiograph of the shoulder showing lateral displacement of the humeral head with respect to the glenoid, losing the half-moon overlap (black arrow) (**a**), compared to a plain radiograph of a normal shoulder, illustrating the half-moon overlap (**b**).

On a normal anteroposterior shoulder X-ray, there is overlap between the humeral head and the glenoid, giving the appearance of a half-moon (Figure 4b) [23]. In cases of posterior dislocation of the shoulder, the lateral and posterior displacement of the humeral head will result in the loss of this sign. Radiographic signs of posterior shoulder dislocation are more subtle than those of anterior shoulder dislocation, and this may delay diagnosis; hence, the importance of this sign (Figure 4a) [24]. In shoulder trauma with limited external rotation, loss of half-moon overlap on the AP view increases suspicion for posterior dislocation and expedites reduction, preventing missed injuries.

### 2.5. Milky Way Sign

#### 2.5.1. On Breast Imaging

**Figure 5:**
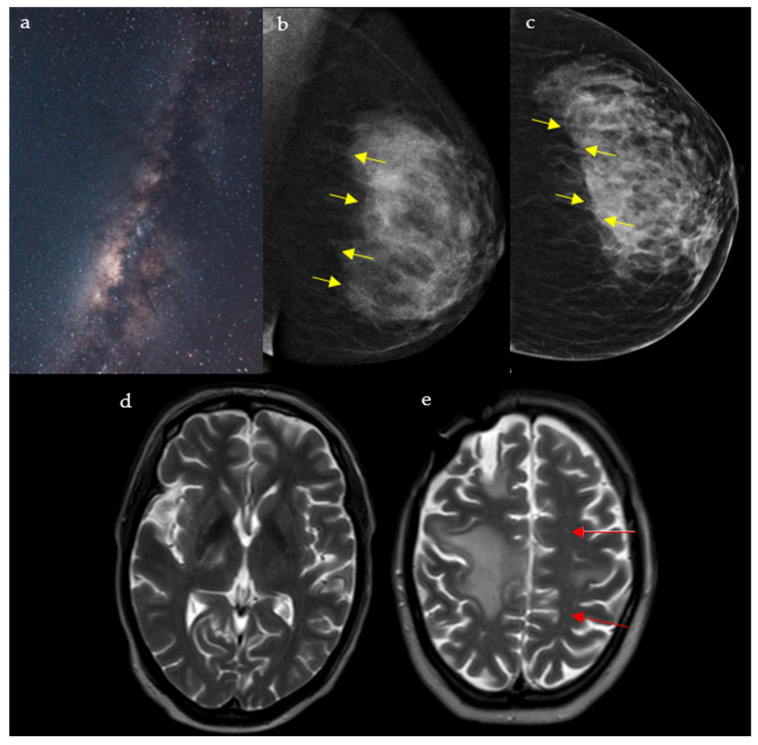
A rendering of the Milky Way galaxy as it appears from space (**a**), resembling the junction between the fibroglandular tissue and the retromammary fat, with an appearance analogous to that of a dark galaxy studded with stars seen on mediolateral oblique (MLO) (**b**) and craniocaudal (CC) (**c**) views of the left breast (yellow arrows). The galaxy also resembles the multiple punctate regions of high T2 surrounding the main component of the lesion in PML (red arrows) (**d**,**e**).

Mostly detected on breast tomosynthesis, the Milky Way (Figure 5a) pattern is one of the signs that help in the diagnosis of ductal carcinoma in situ (DCIS), the most common type of localized breast malignancy, as well as, less frequently, invasive ductal carcinoma (IDC) and atypical ductal hyperplasia (ADH) [25]. It represented linearly arranged microcalcifications distributed along the non-calcified portions of the lactiferous ducts, which give a hazy background appearance. Multiple fine, punctate calcifications aligned in a linear or segmental distribution on mammography raise suspicion for ductal carcinoma in situ and may prompt biopsy in the appropriate clinical setting. In addition, the “Milky Way” is also the name attributed to the retromammary band of fat coursing parallel to the pectoral muscle seen on the mediolateral oblique (MLO) view of a mammogram (Figure 5b,c) [26,27]. This area deserves particular attention and represents one of the check zones in mammographic interpretation [27].

#### 2.5.2. In Neuroimaging

This pattern is seen in cases of progressive multifocal leukoencephalopathy (PML) on the T2/FLAIR sequence of a brain MRI. It appears as punctate hyperintense T2 lesions surrounding the main PML lesion, hence the resemblance to the Milky Way (Figure 5d,e) [28]. PML is a demyelinating disease of the central nervous system caused by the reactivation of the JC virus, seen in immunocompromised patients [29]. Interestingly, the Milky Way pattern differs in aspect depending on the etiology behind PML: A study showed that it is more frequent in natalizumab induced PML, when compared to rituximab or human immunodeficiency virus (HIV) induced disease [29]. Numerous punctate enhancing lesions scattered around a dominant white-matter plaque are highly suggestive of progressive multifocal leukoencephalopathy in immunosuppressed patients presenting with subacute cognitive decline, motor weakness, or visual disturbances, supporting urgent evaluation for JC virus and immune-modulating therapy.

### 2.6. Starfield Pattern 

**Figure 6:**
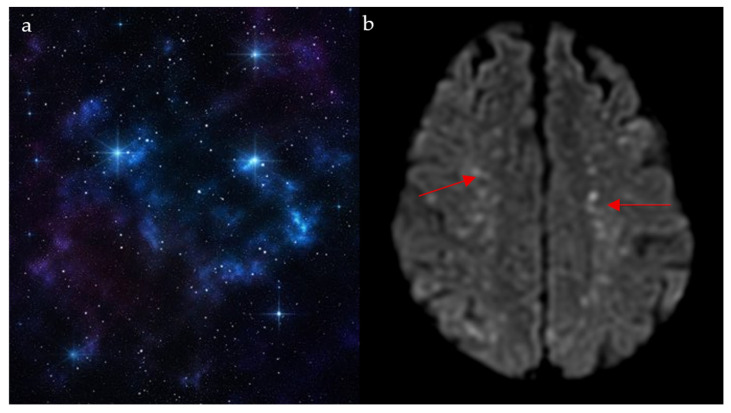
The appearance of a starfield pattern in space (**a**), which holds resemblance to the multiple bright foci on MR diffusion weighted images on axial cuts of the brain (red arrows) (**b**).

This finding is mostly seen with cerebral fat embolism (CFE) seen on magnetic resonance (MR) diffusion-weighted imaging (DWI), and it is usually associated with long-bone fractures [30]. Resembling shining stars in a starfield (Figure 6a), it appears as innumerable non-confluent high-intensity lesions spread throughout the brain parenchyma, involving both the white matter (subcortical and centrum semiovale) and gray matter deep nuclei (Figure 6b) [31]. These lesions represent foci of cytotoxic edema, which develop acutely; hence, their clinical importance in the early diagnosis and management of CFE. It is also important to note that the number of lesions is closely correlated with the Glasgow Coma Scale (GCS) [31]. In patients with a history of long-bone fracture or orthopedic procedures presenting with respiratory distress, petechial rash, and altered mental status, innumerable punctate DWI lesions enable early diagnosis of cerebral fat embolism, prompting supportive critical care.

### 2.7. Star Sign

**Figure 7:**
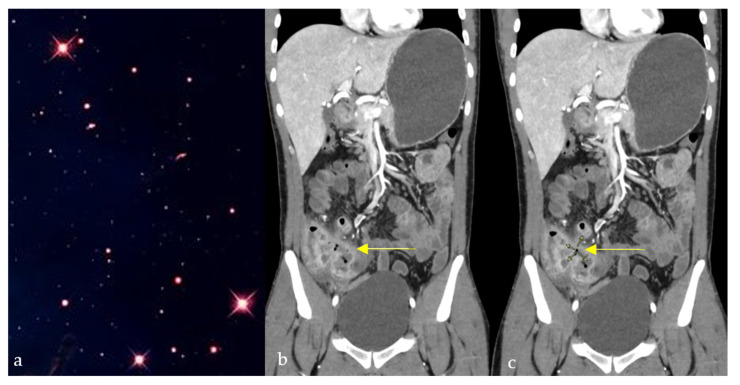
Coronal CT images of the abdomen and pelvis showing a conglomerate of inflamed bowel loops interconnected by multiple fistulous tracts (**b**,**c**), giving a star-shaped configuration (yellow arrows) resembling its natural counterpart (**a**).

The star sign describes the pathognomonic appearance of complex entero-enteric fistulas seen in cases of penetrating Crohn’s disease resembling a shining star (Figure 7a), typically appearing as high T2 and contrast-enhanced T1 tubular structures on magnetic resonance enterography (MRE), or less frequently, on CT enterography [32]. It results from severe inflammation causing ulcerations and multiple fistulous tracts, causing tethering of inflamed and edematous bowel loops, giving a stellate appearance (Figure 7b,c) [33]. The star sign has an estimated sensitivity and specificity of 78.6% and 96.7%, respectively [34]. In Crohn’s patients with abdominal pain, diarrhea, or perianal drainage, a stellate cluster of enhancing fistulous tracts on MRE (“star sign”) indicates a complex fistulizing disease requiring advanced medical or surgical therapy.

### 2.8. Starry Sky Appearance

#### 2.8.1. On Ultrasound

**Figure 8:**
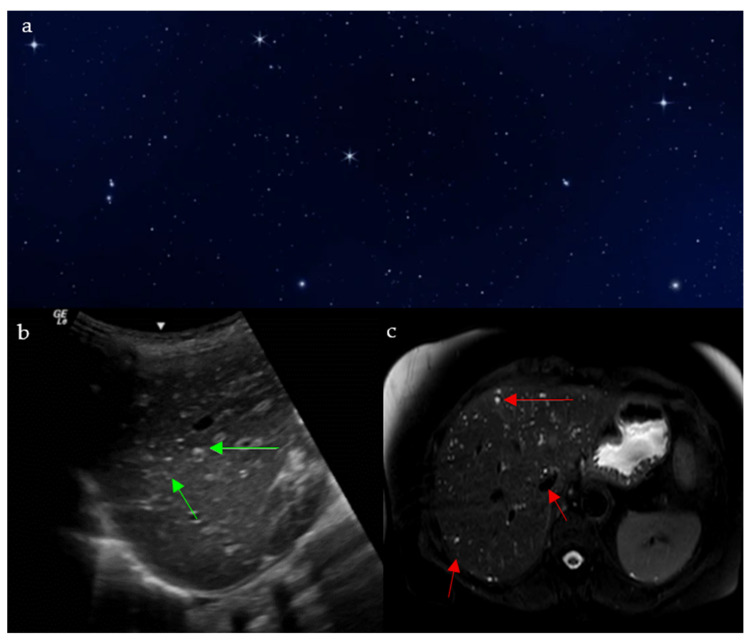
The appearance of a starry sky (**a**), resembling bright echogenic dots throughout a background of decreased liver parenchymal echogenicity on ultrasound (green arrows) (**b**), and small innumerable T2 hyperintense bile duct hamartomas and biliary microhamartomas, scattered throughout the T2 hypointense hepatic parenchyma (red arrows) (**c**).

On sonographic evaluation of the liver, the starry sky (Figure 8a) appearance is indicative of acute viral hepatitis. However, it has low sensitivity and specificity, with one study reporting this sign in only 19 patients out of 791 patients with acute viral hepatitis [35], as similar patterns may be seen in other systemic diseases such as leukemia, lymphoma, toxic shock syndrome, and right-sided heart failure [36,37], and may be even seen on normal liver scans in thin subjects [35]. The diffuse inflammatory disease process causes hepatomegaly with decreased echogenicity of the liver parenchyma, resulting in relative hyperechogenicity of the portal triad and accentuated brightness of the portal vein radicle walls [38]. This consequently gives the recognizable starry sky appearance (Figure 8b) [39].

The starry sky appearance can also be seen on testicular ultrasound, as seen with testicular microlithiasis. Testicular microlithiasis are scattered punctate echogenic foci, representing calcifications within seminiferous tubules [40]. Clinical implications are not clear: some studies showed association with development of testicular tumors [40], others with infertility [41].

#### 2.8.2. On MRI

The starry sky pattern seen in the liver on MRI points towards biliary hamartomas, also called Von Meyenburg complexes [42]. They are indicative of a benign congenital disease caused by an abnormal remodeling of ductal plates during the development of intrahepatic bile ducts [43]. Biliary hamartomas may be associated with nonspecific symptoms such as right upper quadrant discomfort, fatigue, or mild elevations in liver enzymes, though many patients remain asymptomatic until incidental detection. On MRI, it shows as non-enhancing multiple liver nodules that are hypointense on T1 weighted images, and hyperintense on T2 weighted images, with no communication with the bile ducts on MR cholangiography (Figure 8c) [44]. Although a benign pathology, the lesions have the potential of transformation into malignant cholangiocarcinoma, so further follow up imaging is indicated [45].

This sign is also used to describe the appearance of the brain on CT or MRI in cases of neurocysticercosis, which is a parasitic disease caused by *Taenia solium*. Imaging findings vary depending of the stage of the disease [46]. On CT, it can consist of multiple punctate calcified lesions throughout the brain parenchyma. On MRI, multiple hyperintense lesions can be seen on T2-WI, with central hypointensities representing the parasitic scolexes, a finding that is highly suggestive of the diagnosis [47].

### 2.9. Sunburst Appearance and Sign

#### 2.9.1. On Skeletal Imaging

**Figure 9:**
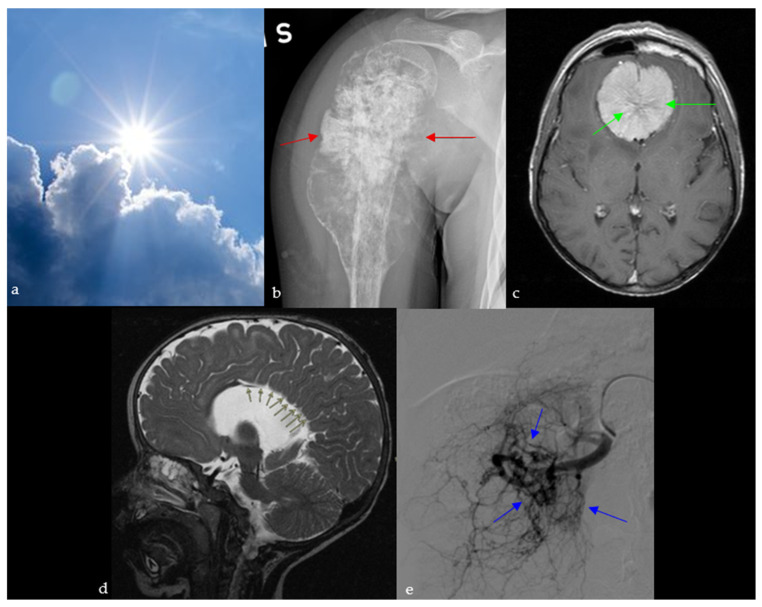
A sunburst appearance observed in nature (**a**), which resembles the aggressive periosteal reaction with stretching of Sharpey’s fibers perpendicular to the humeral bone on plain radiograph (red arrows) (**b**), the spoke wheel pattern of vessels in meningioma viewed in cross-section, diverging from dural attachment on brain MRI (green arrows) (**c**), sagittal T2 images of the brain showing radial orientation of the sulci from the roof of the third ventricle due to dysgenesis of the cingulate gyrus and corpus callosum (green arrows) (**d**), and the appearance of arterial blush seen at selective arterial DSA of a renal angiomyolipoma (blue arrows) (**e**).

Periosteal reactions are caused by the separation of the periosteum from the cortical bone as a result of various pathologies, depending on which a certain radiographic pattern develops [48]. One of those is the sunburst appearance (Figure 9a), a type of spiculated periosteal reactions. The sunburst periosteal reaction in these cases is often associated with localized bone pain, swelling, and reduced joint function in adolescents and young adults. It is typically seen on X-ray, CT, and MR in the setting of conventional osteosarcoma or less frequently Ewing’s sarcoma, which is more known for its perpendicular spicules (Figure 9b) [48,49]. Osteosarcomas are high-grade tumors commonly seen in patients below 25 years old, mostly affecting the fibula, tibia, and femur [48].

It is also used to describe intraosseous hemangiomas, which are benign, slow-growing osseous venous malformations. On plain X-ray and CT, they are seen as osteolytic lesions, with a sunburst trabecular pattern, caused by the thickening of the trabeculae radiating from the center of the lesion [50].

#### 2.9.2. In Neuroimaging

##### Meningioma

The sunburst appearance is a characteristic of meningiomas, which are one of the most common brain neoplasms. Meningiomas are often associated with headaches, seizures, focal neurological deficits, or incidental discovery, reflecting their mass effect and common presentation as slow-growing extra-axial tumors. It can be seen on angiography or brain MRI and is caused by the unique vasculature of these tumors: they are supplied by dural branches of the external carotid and vertebral arteries as well as arteries originating from the pia matter. These vessels diverge from the center of the lesion, giving the sunburst appearance on angiography, T2-WI, or after contrast administration (Figure 9c) [51,52].

##### Agenesis of the Corpus Callosum

Agenesis of the corpus callosum represents a spectrum of congenital abnormalities, with varying severity depending on the time of the triggering insult during fetal life [53]. Agenesis of the corpus callosum is often associated with developmental delay, seizures, and cognitive or motor impairment, reflecting the wide clinical spectrum of this congenital anomaly. It is associated with absence of the pericallosal and cingulate sulci as well as multiple other abnormalities, including sulcal and infratentorial anomalies [54,55]. On midline sagittal images on ultrasound, MRI or CT, the sulci of the medial surface of the hemispheres appear to be radially aligned originating from the roof of the third ventricle, giving a sunburst appearance (Figure 9d) [54].

#### 2.9.3. On Abdominal Imaging

Here, the sunburst sign describes the abnormal branching arteries containing a multitude of aneurysms on the nephrographic phase of renal angiography of an angiomyolipoma, and occasionally on the arterial phase of an abdominal CT scan (Figure 9e) [56]. This benign tumor is the most common mesenchymal mass seen in kidneys, and it originates from abnormal growth of epithelioid cells located around blood vessels. They can be sporadic (usually solitary) or part of a syndrome such as tuberous sclerosis (multiple and bilateral). In this case, the patient presented with flank pain and hematuria, with renal angiography revealing the sunburst vascular pattern characteristic of a solitary angiomyolipoma.

### 2.10. Twinkling Artifact

**Figure 10:**
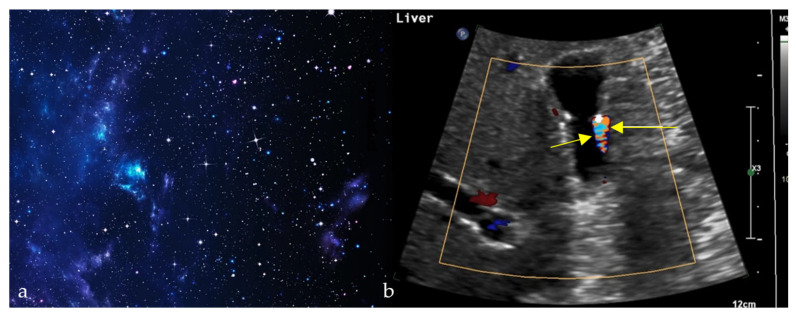
Alternating colors on Doppler signal behind a reflective object (such as a calculus) (yellow arrows) (**b**), giving the appearance of turbulent blood flow and resembling a twinkling sky (**a**).

Often associated with the color comet-tail sign and resembling a twinkling sky (Figure 10a), this artifact comprises rapidly alternating mixtures of colorful Doppler signals on ultrasound and is thought to be caused by irregular reflective surfaces (Figure 10b) [57]. In a patient with flank pain and hematuria, the twinkling artifact on Doppler ultrasound can reveal stone fragments in nephrolithiasis. However, this sign’s clinical uses are wide, proving most useful in enhancing subtle abnormalities on gray scale ultrasound [57]. A recent meta-analysis of 16 studies, including 4572 patients, reported a pooled sensitivity of 86% and specificity of 92% for the Doppler twinkling artifact, supporting its use as a complementary tool for the diagnosis of urolithiasis [58]. It also has a role in detecting microcalcifications on breast, thyroid, and tissue ultrasound [57].

### 2.11. Crescent Sign

#### 2.11.1. On Pyelography

A patient presenting with flank pain, nausea, and recurrent urinary tract infections may show a thin peripheral crescent of contrast on corticomedullary-phase CT, reflecting severe hydronephrosis from obstruction. In such cases, the increased pressure in the urinary collecting system causes the collecting tubules to be compressed, and hence, change their spatial organization to become parallel to the renal convexity [59]. During the corticomedullary phase of an enhanced CT, this will appear as thin crescentic contrast material at the periphery of the kidney that will soon disappear as the pelvicalyceal system becomes opacified [60].

#### 2.11.2. On Conventional Radiographs

**Figure 11:**
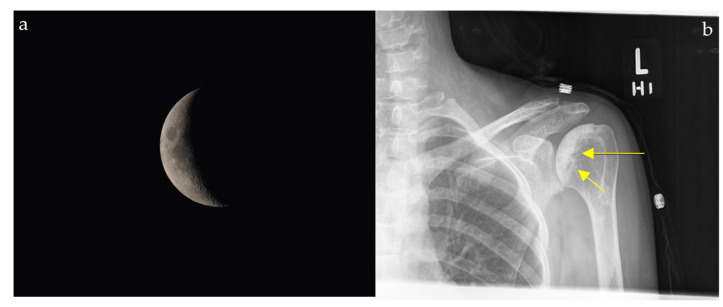
The appearance of a waxing gibbous, soon to be a crescent moon (**a**) resembling a linear cleft due to subchondral fracture in the setting of osteonecrosis seen on plain shoulder radiograph (yellow arrows) (**b**).

A patient with progressive groin pain, limp, stiffness, and reduced hip mobility may demonstrate a subchondral radiolucent crescent on radiographs resembling a crescent moon (Figure 11). On conventional radiographs, the crescent sign is a subchondral radiolucent stripe that typically characterizes stage III avascular necrosis of the femoral head (Figure 11b) [61]. It is caused by the gradual collapse of the dead subchondral trabeculae resulting from repetitive cycles of osteonecrosis and repair. Many disease processes can lead to AVN, such as states of hypercortisolism, diseases of the hemoglobin chains, trauma, kidney disease, radiation, and others [61].

### 2.12. Hyperdense Cresent Sign

**Figure 12:**
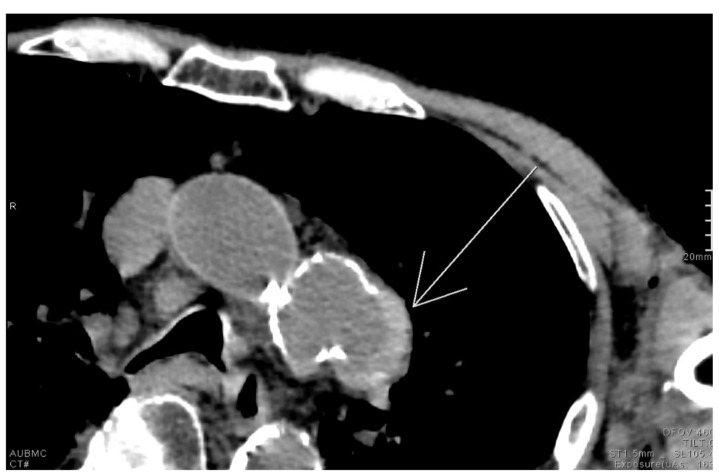
Unenhanced chest CT with findings of the hyperdense crescent sign noted along the left lateral wall of a saccular aneurysm arising from the distal aortic arch (white arrow).

The hyperdense crescent sign, also known as the high-attenuation crescent sign, represents the accumulation of blood within an intraluminal thrombus or the wall of an aortic aneurysm. The sign is considered a warning of aneurysm instability and indicates a higher risk for imminent rupture. The sign is best seen on non-contrast CT scans of the chest or abdomen and develops due to the hyperattenuating nature of fresh blood compared to older thrombus or soft tissue (Figure 12). Patients with abdominal or back pain, hypotension, or a pulsatile abdominal mass who show a hyperattenuating crescent within the aortic aneurysm wall on non-contrast CT are at high risk of impending rupture. This finding signals wall instability and mandates urgent vascular surgery consultation and close hemodynamic monitoring [62].

### 2.13. Lateral Crescent Sign

**Figure 13:**
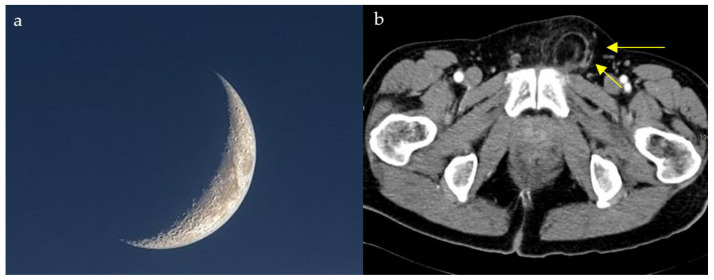
Compression and lateral displacement of the inguinal canal contents by the hernia to form a semicircle of tissue seen lateral to the hernia on axial CT images of the pelvis (yellow arrows) (**b**), resembling a crescent moon (**a**).

Distinguishing direct and indirect inguinal hernias on CT scan can be challenging due to the intricate anatomy at the level of the inguinal area. The lateral crescent sign is seen in the setting of direct hernias and is, therefore, helpful for making this distinction. Direct inguinal hernias project through the Hesselbach triangle, an area bordered by the lateral edge of the rectus muscle medially, the inguinal ligament inferiorly, and the inferior epigastric vessels laterally. The compression of the inguinal canal content as well as fat gives the appearance of a characteristic flattened crescent shaped structure resembling a crescent moon (Figure 13a,b) [63]. In a small comparative CT series, the lateral crescent sign identified direct inguinal hernias, with a sensitivity of 69% and a specificity of 80%, supporting its utility while underscoring the need for validation in larger cohorts [64]. Patients with groin swelling, pain, or symptoms of bowel obstruction may show a lateral crescent of compressed tissue adjacent to a direct inguinal hernia on CT. This finding distinguishes direct from indirect hernias, guiding appropriate surgical repair.

### 2.14. Air Crescent Sign

**Figure 14:**
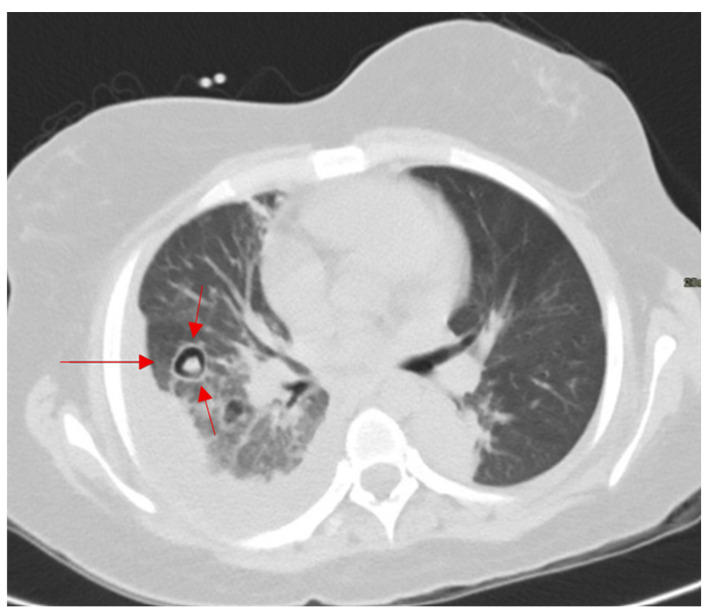
Axial CT of the chest showing a nodular opacity with retracted infarcted lung and crescentic and circular cavitation in pulmonary aspergillosis (red arrows).

In immunocompromised patients with fever, cough, or hemoptysis, an intracavitary air crescent on CT suggests invasive aspergillosis. This sign is one of the delayed signs of invasive pulmonary aspergillosis on conventional chest radiograph or on CT, occurring in approximatively 50% of the patients [65]. It appears encompassed within a pulmonary cavity, and it is the result of the regression of the diseased mass under the effect of neutrophils, indicating bone marrow recovery in immunosuppressed patients (Figure 14) [66]. This finding can also be found in tuberculosis, hydatid cysts, abscesses, carcinomas, and *Pneumocystis carinii* pneumonia [67,68].

To highlight the differences between the various crescent signs described, we provide a comparative summary of their modalities, underlying pathologies, specificity, and clinical importance (Table 1).

### 2.15. Halo Sign

#### 2.15.1. On Ultrasound

The sonographic halo sign is used in the evaluation of isoechoic and hyperechoic liver lesions to distinguish between malignancies and benign tumors, since both may have comparable echogenicities. Studies showed that this sign is especially helpful in differentiating hepatic metastases from hemangiomas, as metastatic lesions are often surrounded by an extra-tumoral hypoechoic halo mostly caused by peritumoral cell compression [69,70].

A similarly named sign is also seen on ultrasound evaluation for large vessel arteritis, such as giant cell arteritis, where the intima media appears thicker than 1 mm due to wall inflammation, giving the appearance of a halo [71]. Ultrasound of the temporal and axillary arteries is recommended as the first-line imaging modality in suspected giant cell arteritis, with pooled sensitivity of 88% and specificity of 96% [72].

#### 2.15.2. On CT

**Figure 15:**
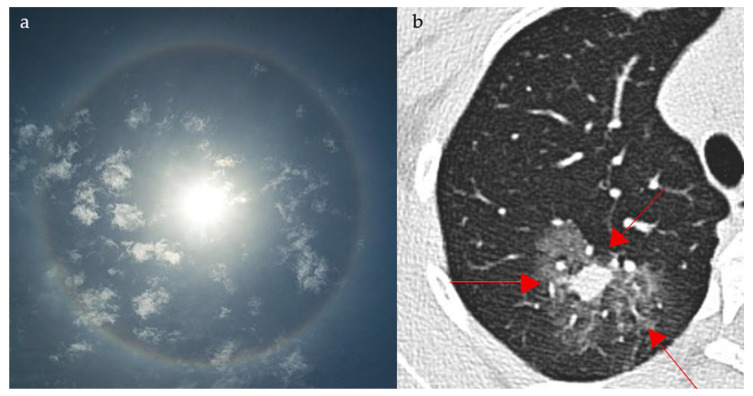
The appearance of a halo surrounding the sun (**a**), resembling a ground glass opacity surrounding a pulmonary nodule, which represents hemorrhage in a case of early pulmonary aspergillosis on axial CT images of the chest (red arrows) (**b**).

The halo sign on chest CT appears, similar to the halo surrounding the sun (Figure 15a), as an area of ground glass opacity (representing alveolar hemorrhage in most cases) surrounding a pulmonary nodule (usually an area of infarction) [73,74]. Diseases causing such a pathological process include early invasive pulmonary aspergillosis, seen in immunocompromised patients (high sensitivity) (Figure 15b), highly vascular metastatic lesions (angiosarcoma, choriocarcinoma, and osteosarcoma), Kaposi sarcoma, vasculitis, and post biopsy changes. Less frequently, it can also be seen in non-hemorrhagic nodules in cases of cancer and inflammation [73]. In COVID-19 pneumonia, the halo sign has also been described as a manifestation of perilesional ground-glass opacity due to alveolar injury and organizing pneumonia, expanding its relevance beyond fungal and hemorrhagic etiologies [75].

An immunocompromised patient presenting with fever, cough, and pleuritic chest pain may demonstrate a halo sign on chest CT, where ground-glass opacity surrounds a pulmonary nodule, most often reflecting early angioinvasive aspergillosis but also seen in hemorrhagic metastases, vasculitis, or post-biopsy change.

### 2.16. Fat Halo Sign

**Figure 16:**
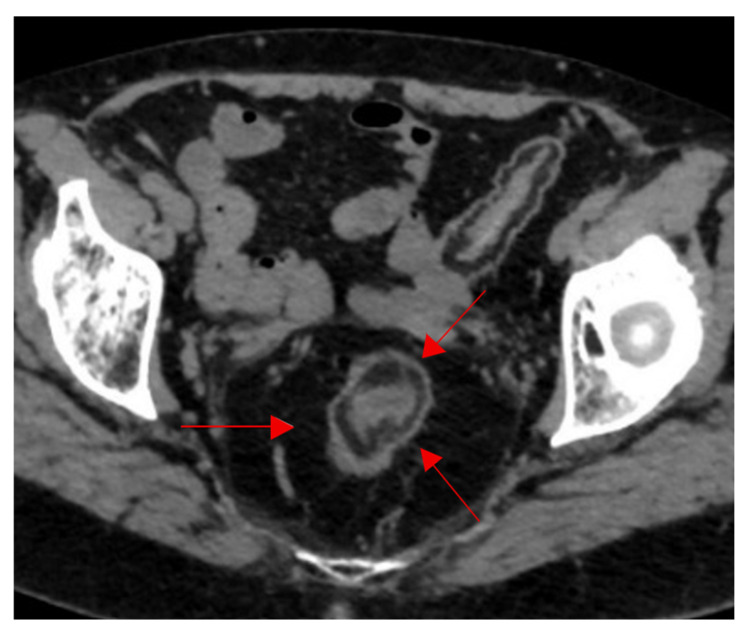
Fat halo sign in longstanding ulcerative colitis. Infiltration of the submucosa with fat between the muscularis propria and the mucosa at the level of the sigmoid colon on axial CT images of the pelvis (red arrows).

Going from inner to outer, the bowel is composed of four major layers, the mucosa, submucosa, muscularis, and serosa, which all normally have soft tissue attenuation on CT images. When the submucosa in infiltrated by fat, it appears thickened with a low density and attenuation usually ranging between −18 to −64 Hounsfield unit (HU), separating the surrounding layers and demonstrating the fat halo sign (Figure 16) [76]. This sign was initially described as characteristic for chronic inflammatory bowel disease, and in this case, it is illustrated in a patient with long-standing ulcerative colitis presenting with chronic diarrhea, abdominal cramping, and rectal bleeding. However, it has a low specificity, as it can be seen in other cases, including cytoreductive therapy, graft versus host disease, or obesity [76,77,78]. Recent data suggest that submucosal fat deposition, often presenting as a fat halo sign, may be linked to visceral adiposity and metabolic risk factors, such as hypertension, diabetes, and elevated vascular calcium scores, reinforcing the need to interpret this sign in the context of metabolic comorbidities rather than solely as an indicator of inflammatory bowel disease [79].

### 2.17. Reversed Halo Sign (Atoll Sign)

**Figure 17:**
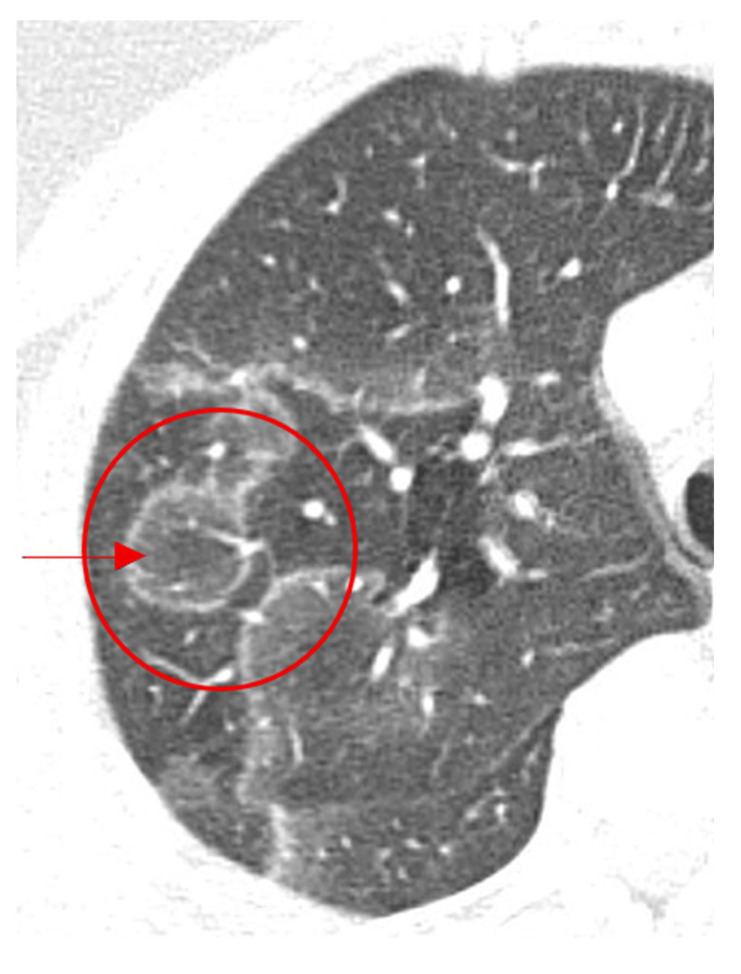
Axial images of the chest showing central ground-glass opacity surrounded by denser air-space consolidation in the shape of a crescent or a ring in organizing pneumonia (red arrow and circle).

The atoll sign appears as a hyperdense ring, representing inflammation in the alveolar ducts, surrounding an area of ground glass opacity attributed to septal inflammation and alveolar cellular debris on chest CT (Figure 17) [80]. It was originally described in the setting of organizing pneumonia, but it was later found to be associated with several pathologies, including Wegener’s granulomatosis, sarcoidosis, and fungal pulmonary infections, notably mucormycosis as well as others [74,81]. In COVID-19 pneumonia, the reversed halo sign may arise not only in organizing pneumonia but also in pulmonary infarction—particularly the so-called “reticular RHS”—where central low attenuation or reticulation, often accompanied by pleural effusion and elevated D-dimer, signals infarction and indicates the need for consideration of CT pulmonary angiography [82]. As such, a peripheral consolidation ring around central ground-glass opacity suggests organizing pneumonia but can also reflect vasculitic, fungal, or viral etiologies; clinical/immunologic context is essential.

The halo signs are contrasted in a separate table to emphasize their differing imaging appearances, associated pathologies, and diagnostic relevance (Table 2).

## 3. Conclusions

Pattern recognition of various pathological conditions is a skill that is harbored with experience and a cornerstone of radiological interpretation. We underscore that these signs are presented as educational pattern-recognition aids rather than validated diagnostic tests; future prospective studies, including trainee assessments and diagnostic accuracy analyses, are warranted to quantify their impact. Recognition of these patterns is relevant across resource settings—from low-resource environments, where early pattern recognition may be useful when advanced imaging or subspecialty consultation is limited, to tertiary centers, where signs augment teaching and refine differential diagnosis.

It is also important to recognize that the reliability of radiological signs is influenced by both the imaging modality and observer factors. Ultrasound-based signs, for example, may at times represent artifacts and are inherently more operator-dependent, whereas CT and MRI tend to yield more reproducible findings. Furthermore, inter-observer variability exists, with interpretation occasionally differing between readers. For this reason, imaging signs should always be considered in conjunction with the clinical presentation and ancillary imaging features rather than in isolation.

This pictorial review provides a comprehensive set of classic radiologic manifestations that resemble various cosmic sights. Such patterns are highly memorable for the trained radiologist and can thus facilitate easier recognition of pathologies and confident diagnosis.

## Figures and Tables

**Table 1:** Comparative summary of crescent-related signs.

Sign	Modality	Typical Pathology	Specificity	Clinical Importance
Crescent Sign	Contrast-enhanced CT	Severe hydronephrosis	Moderately specific when seen in corticomedullary phase; transient	Indicates severe obstruction; must be correlated with other signs of hydronephrosis for confirmation.
AVN Crescent Sign	Radiograph/MRI	Stage III avascular necrosis of femoral head	Highly specific for subchondral collapse	Predicts imminent structural failure of femoral head; crucial for staging and surgical planning.
Hyperdense Crescent	Non-contrast CT	Abdominal aortic aneurysm (AAA)	High specificity for aneurysm wall instability	Reliable harbinger of impending rupture; mandates urgent vascular consultation.
Lateral Crescent	CT pelvis	Direct inguinal hernia	Specific for distinguishing direct vs. Indirect hernia	Helps guide surgical approach and repair strategy.
Air Crescent Sign	CT chest	Invasive pulmonary aspergillosis (IPA), cavitary lung disease	Seen in ~50% of IPA cases; not exclusive	Often coincides with neutrophil recovery in IPA, improving prognosis; must be interpreted with clinical and microbiological data.

**Table 2:** Comparative summary of halo-related signs.

Sign	Modality	Typical Pathology	Specificity	Clinical Importance
Halo Sign (CT chest)	CT (thorax)	Early invasive pulmonary aspergillosis, hemorrhagic metastases, vasculitis, COVID-19 pneumonia	Sensitive but not specific; overlaps with infection and inflammation	Recognized early markers in immunocompromised patients; prompts antifungal therapy but requires correlation with clinical/lab data.
Halo Sign (Ultrasound, GCA)	Ultrasound (temporal and axillary arteries)	Giant cell arteritis (arterial wall inflammation)	High specificity when intima-media thickening ≥ 1 mm (sensitivity 88%, specificity 96%)	First-line imaging finding for suspected GCA; can obviate need for temporal artery biopsy in many patients.
Fat Halo Sign	CT abdomen	Chronic inflammatory bowel disease (esp. Crohn’s, UC); also obesity, cytoreductive therapy	Low specificity; may also be seen in non-IBD contexts	Suggests chronicity of bowel inflammation; must be interpreted cautiously, not diagnostic on its own.
Reversed Halo (Atoll) Sign	CT chest	Organizing pneumonia; also vasculitis, fungal infections, COVID-19 pneumonia	Moderate specificity for organizing pneumonia	Expands differential diagnosis; important radiologic clue but requires clinical integration and follow-up imaging.

## Data Availability

Data sharing is not applicable to this article as no new data were created or analyzed in this study. Illustrations of the corresponding sign are royalty-free, free-stock images available for use.
